# Supplementary material for: Prediction of nasal spray drug absorption influenced by mucociliary clearance
Source: PLoS One. 2021 Jan 28;16(1):e0246007. doi: 10.1371/journal.pone.0246007 (PMC7842989; doi:10.1371/journal.pone.0246007)
Supplement: Attachment — Submitted filename: Inthavong2020_Review.pdf [file pone.0246007.s001.pdf]

Review of the manuscript PONE-D-20-28395, titled "Prediction of nasal spray drug absorption influenced by mucociliary clearance" by Yidan Shang, Kiao Inthavong, Dasheng Qiu, Narinder Singh, Jiyuan Tu
(under consideration for publication at PLOS ONE)

This work uses CFD-based approach to quantify regional drug deposition inside a nasal geometry and tracks the subsequent mucociliary transport of the deposited particles. The study then uses 1-D diffusion model to predict absorption of drug solute into the gel-like mucus upper layer. I have the following comments and questions for the authors:

1. This is a very interesting study and can provide a more realistic picture on therapeutic transmission to the epithelial cells, which can have wider clinical ramifications.

2. The authors should add more details on how the mucus source term was determined. Was it through a series of iterative simulations, so as to achieve the stated 6 mm/min averaged mucus velocity along the main cavity walls?

3. On page 10, the authors state that "the particles were assumed to immediately dissolve into macromolecules after depositing". I presume the "macromolecules" constitute the solute agents that are now on the mucus upper layer. At time t = 0, what was the area over which the solute agents were concentrated? And how does it relate to the size of the particle / droplet that delivered those solute agents?

4. What is the solute concentration in the drug particles / droplets? Is there a distribution for the 1-110 nm solute radii that are embedded / suspended in each droplet? These details seem to be missing in the manuscript.

5. I understand that an experimental validation of the solute transport trends might be out-of-scope for this manuscript. But it would be useful to the reader if the authors can elaborate on the possible validation approaches, as part of the discussion section.

6. Could you label Stream 1, Stream 2 etc. on Figure 4b? This is in context to the comments on page 4, second paragraph.

7. There are some typos in the manuscript. E.g. (a) in the abstract: "The spray particle deposition distribution was validated experimentally and numerically, and the mucus velocity field"; (b) on page 14: "The RSM The simulation was considered converged..."

In conclusion: I think the manuscript, as it is now, needs some minor revisions.
